# Mandibular reconstruction with fibula free flap: a comparative analysis of clinical outcomes of CAD/CAM-assisted surgery versus model-assisted conventional technique

**DOI:** 10.1007/s10006-026-01630-9

**Published:** 2026-08-26

**Authors:** C. Giraudi, Ylenia Gugliotta, F. Secco, A. Novaresio, M. Fasolis, G. Ramieri

**Affiliations:** Maxillo-Facial Surgery Unit, Department of Surgical Sciences, AOU Città della Salute e della Scienza, University of Turin, Corso Bramante 88, 10126 Torino, Italy

**Keywords:** Free fibula flap, Mandible reconstruction, CAD-CAM surgery, Virtual planning, In-house planning

## Abstract

**Objectives:**

The aim of this study was to compare clinical and patient-reported outcomes of mandibular reconstruction with fibula free flap (FFF) performed using model-assisted conventional technique versus computer-aided design/computer-aided manufacturing (CAD/CAM) with virtual surgical planning (VSP).

**Materials and methods:**

Patients who underwent microvascular mandibular reconstruction with FFF between January 2016 and December 2023 were retrospectively reviewed. Inclusion criteria were age ≥ 18 years, segmental mandibular resection, and a minimum follow-up of 12 months. Patients were divided into two groups: model-assisted conventional reconstruction (Group A) and CAD/CAM-assisted reconstruction with VSP (Group B). Demographic, pathological, and surgical variables were collected. Morphological and functional outcomes were assessed using validated instruments including the Katsuragi Scale, FACE-Q Aesthetics, EORTC QLQ-H&N43, MD Anderson Dysphagia Inventory (MDADI), and Speech Handicap Index (SHI).

**Results:**

Thirty-two patients were included (11 in Group A, 21 in Group B). CAD/CAM-assisted reconstruction showed significantly better aesthetic outcomes according to both patient and surgeon evaluations with Katsuragi Scale (*p* = 0.0001). FACE-Q Satisfaction with Surgical Outcome scores were significantly higher in Group B (*p* = 0.02). Patients treated with CAD/CAM also reported significantly fewer self-reported speech-related disturbances according to the SHI (*p* = 0.01). Other functional measures showed favourable trends for CAD/CAM reconstruction but did not reach statistical significance.

**Conclusions:**

CAD/CAM-assisted reconstruction was associated with improved aesthetic outcomes, greater patient satisfaction, and better speech-related function compared with conventional techniques.

**Clinical relevance:**

Digital surgical planning may improve the precision and predictability of mandibular reconstruction and was associated with better patient-reported outcomes, particularly in speech-related function.

## Introduction

Mandibular resection is indicated for a wide range of conditions, including malignant and benign tumours, traumatic injuries, and osteonecrosis [1]. First introduced by Hidalgo et al. in 1989 [2], the fibula free flap (FFF) has become the gold standard for reconstruction following extensive mandibular resections, owing to its relative ease of harvest, favourable bone quality, and adequate pedicle length [3–5].

Successful mandibular reconstruction requires careful consideration of multiple factors to achieve satisfactory outcomes. Both aesthetic and functional aspects must be addressed, in addition to complete oncological resection. Restoration of facial aesthetics encompasses the contour, projection, width, and length of the mandible, while functional reconstruction is essential for respiration, swallowing, and speech [1].

Historically, these reconstructions relied on conventional techniques, often involving intraoperative flap modelling and manual plate contouring, which could contribute to variability in outcomes. Although effective in many cases, conventional approaches are technically demanding, with a steep learning curve, and may yield less predictable results in terms of accuracy, symmetry, and functional performance [6, 7].

Over recent decades, advances in digital technologies have led to the development and adoption of computer-aided design and computer-aided manufacturing (CAD/CAM) in reconstructive surgery [8, 9]. These approaches incorporate virtual surgical planning (VSP) and enable the fabrication of patient-specific cutting guides and pre-bent or custom-made reconstruction plates.

Numerous authors have demonstrated that the use of VSP in FFF mandibular reconstruction enhances precision, efficiency, and predictability, while improving control over functional outcomes and reducing operative time and complication rates [10, 11].

The aim of the present study was to compare CAD/CAM-assisted and conventional fibula free flap mandibular reconstruction techniques in terms of long-term patient-reported aesthetic, functional, and quality-of-life outcomes, using a comprehensive set of validated assessment instruments.

## Materials and methods

### Study design and patient selection

Patients who underwent FFF mandibular reconstruction between 1 January 2016 and 31 December 2023 were retrospectively enrolled in this study.

The inclusion criteria were as follows: (1) primary or secondary mandibular reconstruction following segmental mandibular resection; (2) age ≥ 18 years; (3) minimum postoperative follow-up of 12 months; (4) complete clinical and radiological documentation.

Patients who underwent concomitant tongue resection or experienced partial or total flap loss postoperatively were excluded, as their clinical outcomes could not be reliably evaluated due to potential confounding from the extent of tongue resection and the sequelae of flap failure, respectively.

Patients were allocated into two groups according to the surgical technique adopted: Group A comprised patients treated with model-assisted conventional technique, whereas Group B included patients who underwent surgery assisted by virtual surgical planning (VSP).

Data were retrieved from electronic medical records, operative reports, histopathological documentation, and follow-up records from the Maxillofacial Surgery outpatient clinic of the University of Turin. The variables collected included demographic characteristics (age and sex), lesion pathology, type of mandibular resection (according to the Brown classification) [12], type of reconstruction (osseous, osteocutaneous, or osteomyocutaneous flap), number of fibular segments, use of cutting and repositioning guides, operative time, postoperative chemotherapy and/or radiotherapy, and postoperative complications.

Eligible patients were contacted and invited to undergo a comprehensive clinical evaluation, including detailed medical history and physical examination. Clinical and morpho-functional outcomes were assessed using objective parameters together with validated patient- and surgeon-reported outcome measures. 

### Questionnaires and outcome measures

Subjective morphological and functional outcomes were evaluated using several validated instruments.

The Katsuragi Scale [13, 14] was used to assess facial symmetry, chin projection, and the presence of facial scars; both patients and surgeons independently completed the scale to evaluate postoperative aesthetic conditions. The scoring system comprises four categories of assessment: excellent (symmetrical mandibular contour with no visible scarring); good (mild asymmetry without visible scarring); fair (mild asymmetry with a visible skin graft but no dyschromia); and poor (asymmetry or inadequate chin projection associated with skin dyschromia) [13, 14]. Two modules of the FACE-Q Aesthetics© Scale were administered (Satisfaction with surgical outcomes and Psychological well-being) to assess patient-reported outcomes; the total score is converted to a scale from 0 to 100 where higher scores reflect a better outcome [15, 16]. Quality of life was evaluated using the EORTC QLQ-H&N43 questionnaire, which investigates chewing, swallowing, speech, pain, and psychological status in head and neck cancer patients. All items have a Likert-like response format (not at all = 1; a little = 2; quite a bit = 3; and very much = 4); scores for all scales and single items are calculated by linear transformation of raw scores into a 0–100 score, with 100 representing heavy symptom burden [17]. Swallowing function was further assessed using the MD Anderson Dysphagia Inventory (MDADI); composite score ranges from 20 (extremely low functioning) to 100 (high functioning) [18]. Speech performance was evaluated with the Speech Handicap Index (SHI), which examines voice intensity, vocal fatigue, effort required for speech, and intelligibility; the score ranges from 0 (excellent) to 100 (poor) [19].

### Statistical analysis

Categorical variables were compared between Group A and Group B using the chi-square test, with the significance level set at 5%. When expected frequencies in contingency tables were small and the assumptions of the chi-square test were not met, Fisher’s exact test was applied. The distribution of continuous variables was assessed using the Shapiro–Wilk test. Questionnaire scores were primarily compared between groups using Student’s *t*-test, except for the Katsuragi scale, which was treated as a categorical variable. Because the Speech Handicap Index (SHI) showed a markedly skewed distribution with a high proportion of zero values, a Mann–Whitney U test was additionally performed as a sensitivity analysis to assess the robustness of the findings. In addition, effect sizes (Cohen’s *d*) and 95% confidence intervals for mean differences were calculated to quantify the magnitude and precision of the observed effects. All statistical analyses, including descriptive and inferential statistics, were performed using Microsoft Excel (Microsoft Corp., Redmond, WA, USA). 

### Surgical procedure

All patients underwent preoperative high-resolution computed tomography (CT) of the head and neck, together with lower limb angio-CT to assess vascular anatomy. Axial images were acquired with a slice thickness of 1 mm and a reconstruction interval of 1 mm. All procedures were performed using a two-team approach, allowing simultaneous tumour resection and fibula flap harvesting. The fibula flap was elevated through a lateral approach, and the vascular pedicle was preserved until completion of the resection phase and preparation of the recipient cervical vessels for microvascular anastomosis.

**Fig. 1:**
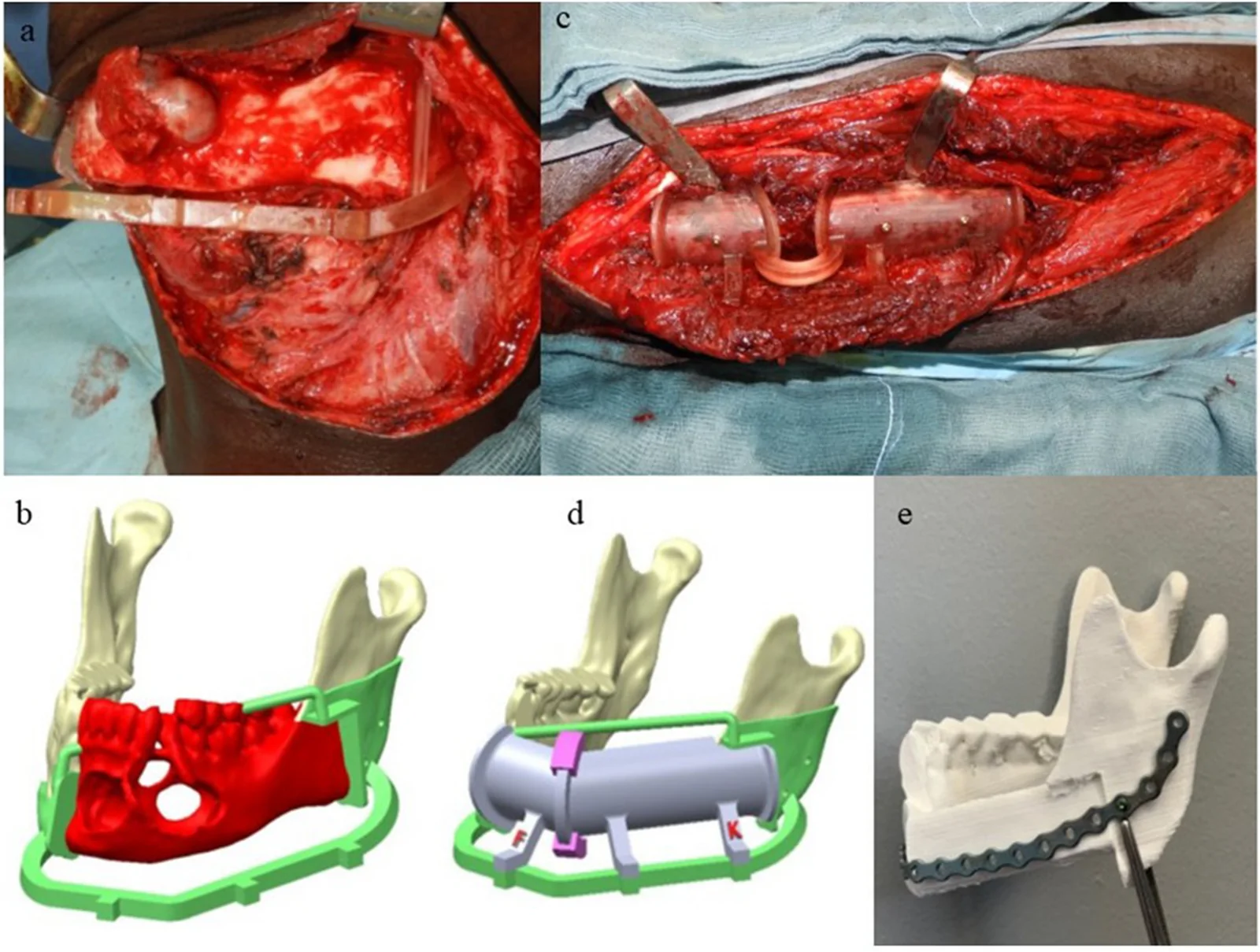
Cutting and repositioning guide design and application. 1**a** mandibular cutting guide secured to the mandible prior to tumour resection. 1**b** digital design of the mandibular cutting guide. 1**c** fibular cutting guide fixed to the donor site before osteotomies. 1**d** combined cutting and repositioning guides featuring predefined segmentation landmarks and a curved connecting arm, sectioned intraoperatively after flap harvest to allow accurate folding and adaptation of the fibular segments. 1**e** reconstruction plate pre-modelled on a patient-specific three-dimensional printed resin reproduction of the mandible

In cases where cutting guides were employed, the mandibular resection guide was secured to the mandible prior to osteotomies. Following tumour resection, the guide was maintained in position to define the residual bone defect and to provide reference landmarks for subsequent fibular inset (Fig. 1a and b). The fibular cutting guide was similarly fixed to the fibula with screws before osteotomies and remained attached until transfer to the mandibular defect (Fig. 1c). In multi-segment reconstructions, the fibular guide incorporated predefined segmentation landmarks and a curved connecting arm, which was sectioned intraoperatively after flap harvest to enable accurate modelling and folding of the bone segments. The fibula, maintained in association with its cutting guide, was transferred to the mandibular defect using corresponding reference points on the mandibular and fibular guides (Fig. 1d). Fixation was achieved with a pre-shaped reconstruction plate, pre-bent on a stereolithographic model the day prior to surgery (Fig. 1e). Virtual surgical planning (VSP), including in-house design and three-dimensional printing of cutting guides, was performed according to the protocol previously described by Zavattero et al. [20].

In patients who underwent surgery with model-assisted conventional technique, a preoperative mandibular CT scan was obtained and the mandible was segmented. An .STL file was generated and 3D-printed to produce a physical model. A reconstruction plate was then pre-bent on the model the day prior to surgery.

This was a retrospective observational study. The Institutional Research Ethics Committee of Città della Salute e della Scienza di Torino reviewed the study protocol and confirmed that formal ethical approval was not required, as the study involved retrospective analysis of routinely collected clinical data and did not include any intervention beyond standard clinical care. Written informed consent was obtained from all participants. Written informed consent for the publication of clinical photographs was obtained from the patient depicted in Fig. 1.

## Results

Among the 55 patients retrospectively identified based on the inclusion and exclusion criteria, 12 had died, seven were lost to follow-up and four declined to participate.

A total of 32 patients were included in the study; 11 underwent reconstruction using the model-assisted conventional technique (Group A), whereas 21 were treated with the assistance of VSP and CAD/CAM technologies (Group B).

Table 1 summarizes the patient characteristics, pathological findings, and surgical details for the two groups.

**Table 1:** Patient characteristics, pathological findings, and surgical details for the two groups. α. Fisher’s exact test was used

Variable	Total *N*(%) TOT = 32	Group A *N*(%) TOT = 11	Group B *N*(%) TOT = 21	pValue
Gender				0.14 ^α^
Male	18 (56.25)	4 (36.4)	14 (66.7)	
Female	14 (43.75)	7 (63.6)	7 (33.3)	
Histology				
Squamocellular carcinoma	19 (59.4)	6 (54.5)	13 (61.9)	0.19^α^
Ameloblastoma	5 (15.6)	0	5 (23.8)	
Osteonecrosis	3 (9.4)	2 (18.2)	1 (4.8)	
Odontogenic myxoma	2 (6.25)	1 (9.1)	1 (4.8)	
Osteosarcoma	1 (3.1)	1 (9.1)	0	
Ossifying fibroma	1 (3.1)	1 (9.1)	0	
Odontogenic keratocyst	1 (3.1)	0	1 (4.8)	
Type of resection according to Brown [12]				0.05 ^α^
Class 1 (Lateral defect not including canine or condyle)	14 (43.75)	8 (72.2)	6 (28.6)	
Class 1c (Lateral defect including condyle)	1 (3.1)	0	1 (4.8)	
Class 2 (Hemimandibulectomy including ipsilateral but not contralateral canine or ipsilateral condyle)	11 (34.4)	1 (9.1)	10 (47.6)	
Class 2c (Hemimandibulectomy including condyle)	1 (3.1)	0	1 (4.8)	
Class 3 (Anterior mandibulectomy includes both canines but neither angle)	4 (12.5)	2 (18.2)	2 (9.5)	
Class 4 (Extensive anterior mandibulectomy including both canines and one or both angles)	1 (3.1)	0	1 (4.8)	
Class 4c (Extensive anterior mandibulectomy including both canines and one or both condyles)	0	0	0	
Type of flap				< 0.01
Osteomuscular	20 (62.5)	11 (100)	9 (42.9)	
Osteomyocutaneous	12 (37.5)	0	12 (57.1)	
Number of fibula segments				0.65
1	20 (62.5)	8 (72.7)	12 (57.2)	
2	9 (28.1)	2 (18.2)	7 (33.3)	
> 2	3 (9.4)	1 (9.1)	2 (9.5)	
Concomitant tracheostomy	27 (84.4)	9 (81.8)	18 (85.7)	0.77
Concomitant neck dissection	18 (56.2)	6 (54.5)	12 (57.1)	0.89
Adjuvant radiotherapy	8 (25)	1 (9.1)	7 (33.3)	0.13

Among included patients, 18 were male (56.25%); no significant difference in gender distribution was observed between Group A and Group B (Fisher’s exact test, *p* = 0.14).

The mean age was 48.9 ± 17.4 years among female patients and 61.3 ± 13.9 years among male patients. The overall mean age was 55.9 ± 15.4 years (range 19 to 80 years) with no significant difference (t-test, *p* = 0.55) between Group A (53.7 ± 14.2 years) and Group B (57.0 ± 15.7).

The analysis of histopathological documentation revealed 19 cases of squamous cell carcinoma (59.4%), along with five ameloblastomas (15.6%), three cases of osteonecrosis (9.4%), two odontogenic myxomas, one osteosarcoma, one ossifying fibroma, and one odontogenic keratocyst. There is no statistically significant difference in the distribution of histological diagnoses between Group A and Group B (Fisher’s exact test, *p* = 0.19), even when lesions were grouped as malignant and benign (Fisher’s exact test, *p* = 1).

The majority of patients in Group A (72.7%) presented a type I defect according to the Brown classification [12], whereas in Group B the most frequent defect was type II (47.6%), followed by type I (28.6%). The distribution of mandibular defects approached statistical significance between Group A and Group B (Fisher’s exact test, *p* = 0.05).

All patients in Group A (100%) received osteomuscular flaps, whereas in Group B the majority received osteomyocutaneous flaps (57.1%); the distribution of flap types differed significantly between the two groups (chi-square test, *p* < 0.01).

The majority of patients in both Group A (72.7%) and Group B (57.2%) received a single-segment fibula flap, with smaller proportions receiving two or more segments.

Most patients in both groups underwent concomitant tracheostomy (Group A = 81.8%; Group B = 85.7%) and neck dissection (Group A = 54.5%; Group B = 57.1%).

After surgery, radiotherapy was administered to only eight patients (25% of the cohort), including one patient in Group A (9.1%) and seven patients in Group B (33.3%).

The distribution of the number of fibular segments, as well as the rates of concomitant tracheostomy, neck dissection, and adjuvant radiotherapy, did not differ significantly between the two groups (chi-square test, *p* = 0.65, *p* = 0.77, *p* = 0.89 and *p* = 0.13 respectively).

The questionnaire results are reported in Tables 2 and 3.

**Table 2:** Results of the chi-square test comparing patients’ and surgeon’s evaluations of the final outcome according to the Katsuragi scale between Group A and Group B. Statistical analysis demonstrated that Group B reported a significantly higher proportion of patients with excellent or good outcomes compared with Group A

Questionnaire	Group A (*n*. TOT = 11)	Group B (*n*. TOT = 21)	pValue
Katsuragi scale (patient)			**0.0001**
Excellent	5	14	
Good	0	7	
Fair	3	0	
Poor	3	0	
Katsuragi scale (surgeon)			**0.0001**
Excellent	3	12	
Good	2	9	
Fair	4	0	
Poor	2	0	

**Table 3:** T-test results comparing mean questionnaire scores between Group A and Group B. Statistical analysis demonstrated significantly better outcomes for Group B in the Speech Handicap Index (SHI) and in the FACE-Q Satisfaction with Surgical Outcome domain

Questionnaire	Group A (mean ± SD)	Group B (mean ± SD)	Mean difference (95% CI)	Cohen’s d	pValue
FACE-Q – Satisfaction with surgical outcome	48.6 ± 39.4	75.8 ± 25.2	27.2 (0.6 to 53.8)	0.89	**0.02**
FACE-Q – Psychological well-being	60.7 ± 35.1	74.4 ± 24	13.7 (-11.6 to 39.0)	0.49	0.2
MD Anderson Dysphagia Inventory	44.5 ± 30.3	44.8 ± 27.7	0.3 (− 21.3 to 21.9)	0.01	0.97
EORTC QLQ - H&N43	72.7 ± 33.9	55.9 ± 18.2	-16.8 (-40.6 to 7.0)	0.68	0.07
Speech Handicap Index *	32 ± 40.6	5.8 ± 17.2	-26.2 (-47.3 to 5)	0.96	**0.01**

* Sensitivity analysis using the Mann–Whitney U test confirmed the statistical significance of the difference between groups (*p* = 0.04)

*SD* standard deviation, *CI* confidence interval

Subjective patient self-assessment using the Katsuragi scale revealed that, in Group A, nearly half of the patients (45.5%) considered their outcome to be excellent, whereas the remaining patients rated their result as fair or poor (27.3% and 27.3%, respectively). In contrast, in Group B all patients judged their final clinical outcome as either excellent (66.7%) or good (33.3%). Surgeon evaluations were generally less favourable than patient self-assessments in both groups. However, while in Group A patients were distributed across all four assessment categories (excellent 27.3%; good 18.2%; fair 36.4%; poor 18.2%), in Group B all patients were rated as having achieved either an excellent or good outcome (57.1% and 42.9%, respectively). The chi-square test demonstrated that, in both patient and surgeon evaluations, Group B showed a significantly higher proportion of patients achieving excellent or good outcomes compared with Group A (both *p* = 0.0001; Table 2).

The FACE-Q Aesthetics questionnaire evaluation showed higher average scores for Group B in both scales. In the Satisfaction with Surgical Outcome domain, the mean score was 48.6 in Group A compared with 75.8 in Group B; this difference reached conventional statistical significance (*p* = 0.02). In the Psychological Well-Being domain, the mean score was 60.7 in Group A and 74.4 in Group B, indicating higher levels of satisfaction and psychological well-being among patients in Group B; however, this difference did not reach statistical significance (*p* = 0.2; Table 3).

Similarly, in the EORTC QLQ-H&N43, Group B obtained a lower mean score (55.9 ± 18.2 vs. 72.7 ± 33.9), corresponding to a reduced impact of the reported symptoms on patient quality of life; however, this difference also did not reach statistical significance (*p* = 0.07; Table 3).

In the MD Anderson Dysphagia Inventory, Group B reported a slightly better mean score compared with Group A (44.8 ± 27.7 vs. 44.5 ± 30.3); however, the difference did not reach statistical significance (*p* = 0.97; Table 3).

Finally, the Speech Handicap Index questionnaire showed that more than half of the patients in Group A (6 out of 11, 54.5%) and the majority of those in Group B (18 out of 21, 85.7%) achieved a score of 0, indicating a low incidence of speech-related difficulties in these patients. Patients in Group B reported a lower mean score compared with those in Group A (5.8 ± 17.2 vs. 32 ± 40.6, respectively), reflecting a statistically significant lower incidence of speech-related problems in Group B (*p* = 0.01; Table 3). A sensitivity analysis using the Mann–Whitney U test confirmed the statistical significance of the difference in SHI scores between the two groups (*p* = 0.04).

## Discussion

Mandibular reconstruction with a free fibula flap (FFF) remains the gold standard for restoring mandibular continuity following extensive resections for head and neck tumours [21, 22]. However, the anatomical complexity of the mandible presents significant challenges in achieving optimal aesthetic and functional outcomes [23, 24].

The ability to design and fabricate customised surgical guides enables surgeons to plan resections accurately and to rely on tools that facilitate precise mandibular reconstruction [25–27]. The primary objectives are to reduce operative time, improve surgical precision, and optimise both functional and aesthetic outcomes, as the use of stereolithographic models alone does not alleviate the technical complexity of osteotomies and is insufficient to guarantee predictable execution of the surgical plan [28].

Numerous authors have demonstrated that the introduction of virtual surgical planning (VSP) and CAD/CAM technology can significantly reduce critical operative times by 67.4 to 120 min [6, 21, 22, 29–32]. In particular, a substantial reduction in flap ischaemia time has been observed, as cutting guides allow bone segments to be shaped while the flap remains vascularised at the donor site [33, 34].

Taristano et al. (2018) reported a high level of reproducibility in mandibular reconstruction using CAD/CAM, with a mean deviation of 1 mm between the virtual surgical plan and the actual reconstruction [35]. Similarly, Metzler et al. (2014) compared preoperative VSP with postoperative CT scans obtained one week after surgery and demonstrated that VSP significantly enhanced both accuracy and consistency in mandibular reconstruction [36].

Despite these advantages, several important constraints regarding the use of CAD/CAM technology in mandibular reconstruction have been highlighted in the literature. These include the anatomical variability, the presence of multiple soft tissue paddles, and the potential need for intraoperative modifications of the surgical plan [37]. Furthermore, the benefits of this technology in relatively straightforward reconstructions, such as single-segment fibula flaps, remain debated.

In this study, CAD/CAM technology simplified complex mandibular reconstructions, particularly in cases requiring multiple osteotomies [38]. Preoperative VSP combined with intraoperative 3D-printed cutting guides enables precise osteotomies of both the mandible and fibula at predetermined sites [6, 15].

The defining characteristic of this approach is the accurate reconstruction of the neomandible with reduced reliance on the surgeon’s intraoperative experience, as the osteotomised fibular segments are designed to fit into the mandibular defect in a “puzzle-like” manner [6, 28, 36].

However, from a practical perspective, the implementation of CAD/CAM-assisted reconstruction requires access to dedicated planning software, three-dimensional printing facilities, and personnel trained in virtual surgical planning and guide design. The feasibility and cost-effectiveness of these workflows may vary considerably between institutions depending on the availability of technical expertise and in-house manufacturing resources. Therefore, although CAD/CAM technology offers several clinical advantages, its adoption should be evaluated within the context of each institution’s infrastructure, case volume, and available resources.

Beyond these technical considerations, several studies have investigated long-term clinical outcomes.

Bartier et al. demonstrated through morphometric analyses that VSP improves facial symmetry and allows more accurate reproduction of facial contours, particularly at the condyles and mandibular angles [11]. Similarly, Wang et al. (2016) [33] reported enhanced functional and aesthetic outcomes with CAD/CAM technology and Qayyum et al. (2023) [7] observed marked improvements in quality of life following reconstruction and dental rehabilitation, emphasising the importance of restoring not only anatomical continuity but also functional performance.

Despite the growing body of evidence supporting CAD/CAM-assisted mandibular reconstruction, most previous studies have primarily focused on surgical accuracy, operative efficiency, complication rates, or selected functional outcomes. Comprehensive patient-centred evaluations integrating aesthetic perception, oral function, swallowing ability, speech outcomes, and health-related quality of life remain limited. Furthermore, many published series include heterogeneous follow-up periods, potentially influencing the assessment of long-term outcomes. Therefore, the present study was designed to provide a comprehensive comparison between CAD/CAM-assisted and conventional mandibular reconstruction by simultaneously evaluating multiple validated patient-reported outcome measures, including the Katsuragi scale, FACE-Q, EORTC QLQ-HN43, MDADI, and SHI, in a cohort of patients with a minimum follow-up of 12 months. This approach was intended to provide a more comprehensive assessment of the long-term impact of reconstructive techniques on patients’ quality of life and functional recovery.

The results of the present study suggest that VSP and CAD/CAM technologies provide significant clinical advantages over the traditional model-assisted conventional technique, particularly in terms of patient satisfaction with morphological outcomes, especially facial symmetry. This difference may be attributed to the improved ability to determine the exact length of the fibular segment required and to the simplification of fibular segmentation through the use of cutting guides, which facilitate the achievement of a symmetrical mandibular profile. Furthermore, reconstruction plates can be pre-shaped preoperatively on a stereolithographic model reproducing the ideal mandibular contour with the fibula segment already placed in position, allowing optimal positioning and orientation of the fibular flap to mirror the contralateral side and more accurate plate pre-bending.

Similarly, the FACE-Q questionnaire demonstrated statistically significantly higher scores in Group B in the “Satisfaction with Surgical Outcome” domain, suggesting greater satisfaction with aesthetic appearance among these patients.

Regarding subjective quality of life evaluation, both the FACE-Q “Psychological Well-Being” domain and the EORTC QLQ-H&N43, consistent with previous literature [39–43], showed higher mean scores in Group B; however, these differences did not reach statistical significance in this study. These findings should be interpreted with caution, as quality-of-life outcomes are likely influenced by several confounding factors beyond the surgical planning technique. In particular, the distribution of mandibular defects differed between the two groups, approaching statistical significance (Fisher’s exact test, *p* = 0.05). The majority of patients in Group A (72.7%) presented a type I defect according to the Brown classification, whereas in Group B the most frequent defect was type II (47.6%), followed by type I (28.6%). Since more extensive mandibular defects are generally associated with greater functional impairment and more complex rehabilitation, this imbalance may have influenced postoperative quality-of-life outcomes. Furthermore, Group B included a higher proportion of patients who received adjuvant radiotherapy (33.3% vs. 9.1% in Group A), a treatment known to adversely affect swallowing, xerostomia, speech, and other domains assessed by the EORTC QLQ-HN43. Consequently, the borderline difference observed (*p* = 0.07) may reflect, at least in part, differences in baseline disease severity and adjuvant treatment rather than the reconstructive planning strategy itself. The relatively small sample size further limited our ability to adjust for these confounding factors and to isolate the independent effect of the planning technique on patient-reported quality-of-life outcomes.

With regard to functional outcomes, the MD Anderson Dysphagia Inventory did not demonstrate a statistically significant difference in swallowing ability or food intake between the groups. Previous studies have associated CAD/CAM-assisted surgery with improved chewing and swallowing function [44]; however, it has been emphasised that postoperative mastication and deglutition are influenced not only by the reconstructive technique but also by the extent and location of the mandibular defect. In particular, anterior or extensive mandibular defects represent the strongest independent predictors of chewing impairment and reduced oral intake [41, 42, 44]. In the present study, no statistically significant difference was observed between the groups, possibly due to the exclusion of patients undergoing concomitant glossectomy and the limited sample size.

Finally, patients in Group B reported significantly fewer subjective disturbances in speech articulation. Although this finding may partly reflect improved mandibular morphology, it should also be interpreted in light of potential confounding factors, such as the extent and anatomical location of the resection.

The limitations of this study include its single-centre design, retrospective nature, and relatively small sample size. Furthermore, multiple outcome measures were evaluated without formal correction for multiple comparisons. Therefore, statistically significant findings should be interpreted with caution and considered exploratory until confirmed in larger prospective cohorts.

Although the overall distribution of benign and malignant conditions was comparable between the two groups, quality-of-life outcomes may have been influenced by disease-related and treatment-related factors, particularly adjuvant radiotherapy. The specific effects of oncological treatments, including salivary gland dysfunction, trismus, mucositis, and limited mouth opening, were not separately assessed. In addition, the exclusion of patients who died during follow-up may have introduced survivor bias. All deceased patients had undergone reconstruction for malignant disease, and therefore the final study population may not be fully representative of the original cohort. In contrast, patients lost to follow-up or declining participation presented with heterogeneous underlying pathologies, including both benign and malignant conditions.

Additional potential confounding factors, such as oral health status, smoking habits, and age, were not evaluated. Furthermore, the two study groups were not fully comparable with respect to defect classification, flap composition, and adjuvant radiotherapy. These differences reflect the progressive adoption of CAD/CAM technology over the study period and may have influenced both functional and quality-of-life outcomes independently of the reconstructive planning technique. Therefore, observed differences and trends, particularly for outcomes approaching statistical significance, should be interpreted with caution. Furthermore, no cost analysis was conducted.

Nonetheless, the use of a consistent surgical technique, validated assessment instruments, and the evaluation of clinical outcomes at a minimum of 12 months postoperatively represent notable strengths of this study.

## Conclusion

This study suggests the potential clinical value of CAD/CAM technology in mandibular reconstruction with fibula free flap. CAD/CAM-assisted reconstruction was associated with improved aesthetic outcomes, higher satisfaction with surgical outcome, and lower self-reported speech-related impairment compared with the conventional approach. Although favourable trends were also observed in other quality-of-life and functional measures, these differences did not reach statistical significance.

By combining multiple validated patient-reported outcome measures evaluating aesthetics, oral function, swallowing, speech, and health-related quality of life in a cohort with a minimum follow-up of 12 months, this study provides a comprehensive assessment of long-term patient-centred outcomes following mandibular reconstruction. However, given the retrospective design, limited sample size, and potential confounding factors, these findings should be considered exploratory and warrant confirmation in larger prospective studies.

## Data Availability

The data supporting the findings of this study are available from the corresponding author upon reasonable request. Access to the data is subject to institutional and ethical regulations regarding patient confidentiality.
